# Acute anoxic changes in peripheral nerve: anatomic and physiologic correlations

**DOI:** 10.1002/brb3.347

**Published:** 2015-05-06

**Authors:** Michael Punsoni, Steven Drexler, Thomas Palaia, Matthew Stevenson, Mark M Stecker

**Affiliations:** 1 Department of Pathology, Winthrop University HospitalMineola, New York, 11530; 2 Department of Biomedical Research, Winthrop University HospitalMineola, New York, 11530; 3 Department of Neuroscience, Winthrop University HospitalMineola, New York, 11530

**Keywords:** Anoxia, glucose, peripheral nerve

## Abstract

**Introduction:**

The response of the peripheral nerve to anoxia is modulated by many factors including glucose and temperature. The purposes of this article are to demonstrate the effects of these factors on the pathological changes induced by anoxia and to compare the electrophysiologic changes and pathological changes in the same nerves.

**Methods:**

Sciatic nerves were harvested from rats and placed in a perfusion apparatus where neurophysiologic responses could be recorded continuously during a 16 h experiment. After the experiment, light microscopy and electron microscopy were performed.

**Results:**

Light microscopic images showed mild changes from anoxia at normoglycemia. Hypoglycemic anoxia produced massive axonal swelling while hyperglycemic anoxia produced apparent changes in the myelin. Anoxic changes were not uniform in all axons. Electron microscopy showed only minor disruptions of the cytoskeleton with anoxia during normoglycemia. At the extremes of glucose concentration especially with hyperglycemia, there was a more severe disruption of intermediate filaments and loss of axonal structure with anoxia. Hypothermia protected axons from the effect of anoxia and produced peak axonal swelling in the 17–30°C range.

**Conclusions:**

The combination of hyperglycemia or hypoglycemia and anoxia produces extremely severe axonal disruption. Changes in axonal diameter are complex and are influenced by many factors.

## Introduction

The peripheral nerve can be damaged by many physical stresses including trauma, toxins, and anoxia (Stys 2005). These injuries can be probed with physiological studies (Weigl et al. 1989; Bostock et al. 1994; Kiernan et al. 2002; Stecker et al. 2011, 2013a,b), biochemical studies (Okada and McDougal 1971; Hofteig et al. 1981; Brown et al. 2012; Stavniichuk et al. 2012), and histopathological studies (Def Webster and Ames 1965; Nukada and Dyck 1987; Dyck et al. 1990; Edmonds and Koenig 1990; Waxman et al. 1992) among others. This article has two purposes. The first is to describe the effects that glucose concentration and hypothermia have on the histopathological changes induced in peripheral nerve by anoxia. The second is to correlate the changes seen during continuous neurophysiologic recordings with the histopathologic changes in the same nerves.

Def Webster and Ames (1965) found that it was possible to see mitochondrial swelling as quickly as 3 min into anoxia but this swelling becomes irreversible at roughly 30 min when discontinuities in membranes were noted. Mire et al. (1970) demonstrated that unmyelinated axons undergo a period of focal swelling during the early phases of degeneration. The degree of swelling is greatest around 12 h after the initial insult and subsequently decreased over the next 24 h. The authors concluded that the axonal swelling resulted from the intra-axonal accumulation of ions and water. However, the amount of axonal swelling that can be expected from anoxia is more complex than just osmotic forces because the cytoskeleton in mature nerves (Edmonds and Koenig 1990) resists the swelling tendency and swelling is in part related to the state of ion channels (Waxman et al. 1993). Thus, swelling is not a simple function the osmolarity of the external media, the cytoplasm and the state of ionic pumps. In addition to swelling, there are other histological changes seen such as breakdown of the cytoskeleton (Waxman et al. 1994) and swelling of mitochondria (Def Webster and Ames 1965). Correlating these histologic changes with neurophysiologic changes in the function of the nerve is an important step in creating a more accurate, multidimensional picture of the effects of anoxia on peripheral nerve. Also, exploring the effects of hyperglycemia on the response to anoxia can provide a better understanding of neuropathies that are clearly related to anoxia and hyperglycemia such as critical illness polyneuropathy (Bolton 2005) and possibly diabetic neuropathy (Dyck 1989).

## Materials and Methods

All studies were carried out under a protocol approved by the Winthrop IACUC (Winthrop University Hospital Protocol, WUH-MS#1). Sprague-Dawley rats (Hilltop, Scottdale, PA) were used in all experiments. All rats were male retired breeders. The average age in the hypothermia experiments was 29 weeks (range 25–31) and the average range in the glucose experiments was 31 weeks (range 30–32). Sciatic nerves were dissected, removed from the rat and placed into a perfusion chamber and stimulated using stainless steel needle electrodes.

### Neurophysiologic studies

A full description of the methods is given in previous papers (Stecker and Stevenson 2013; Stecker et al. 2013a,b). The stimulus consisted of paired unipolar pulses separated by 4 msec, each with a 15 mA peak current, a duration of 0.01 msec and an overall pair repetition rate of 5 Hz. Bipolar recordings of the nerve action potential (NAP) were made, digitized at 99 kHz, averaged and stored every 4 sec. Neurophysiologic recordings were made continuously throughout the experiment until the nerve was removed from the perfusion apparatus for fixation.

Two types of experiment were performed. In the first, termed “stability” experiments, the nerves were continuously exposed to oxygenated perfusate. In the other experiments, termed “anoxia” experiments, the nerves were subjected to five 90 min periods of anoxia each of which was followed by a 90 min period of recovery in a fully oxygenated perfusate. Anoxia experiments were of the same total length as the “stability” experiments. In order to compare data from the “anoxia” and “stability” experiments, the average of the neurophysiologic quantities at the times corresponding to the 90 min period that would follow each cycle of anoxia are computed. These are referred to as coming from cycle 1, cycle 2, cycle 3, cycle 4 or cycle 5 even though there is technically no “cycle” in the stability experiments.

The entire experiment was under computer control. The base perfusate was composed of 10 mmol/L HEPES, 110.2 mmol/L NaCl, 17.8 mmol/L NaHCO_3_, 4.0 mmol/L MgSO4, 3.9 mmol/L KCl, 3.0 mmol/L KH_2_PO_4_ 1.2 mmol/L CaCl_2_ as in previous studies. Three dextrose concentrations: 0, 5.5, and 55.5 mmol/L were used in these experiments. The length of the nerve segment studied averaged roughly 1 cm.

In all of the experiments where the glucose concentration was either 0 mmol/L or 55.5 mmol/L, the entire experiment was carried out at 37°C. In the experiments assessing the effect of temperature, the oxygenated perfusate was maintained at 37°C, while the temperature of the deoxygenated perfusate was varied between 5 and 42°C. All of the experiments exploring the effect of temperature were done with a glucose concentration of 5.55 mmol/L.

The NAP peak to peak amplitude, conduction velocity, duration, area under the curve (AUC), and the conditioned stimulus response (CSR) are the primary measured parameters which are abstracted automatically with manual supervision. In order to compare the results from different nerves, the values of each of the abstracted parameters are normalized so that their mean values in the baseline oxygenated state is equal to 1.

### Histopathologic studies

All nerves from which neurophysiologic recordings were made were removed from the perfusion apparatus at the end of the experiment an immediately immersed in a fixative solution.

Tissue for light microscopy was placed in formalin, embedded in paraffin and cut in both longitudinal and cross-sections. Special histologic stains (Luxol-Fast Blue-PAS and trichrome) were used in addition to a routine hematoxylin and eosin stain. Twenty-one nerves subjected to anoxia at different temperatures were cross-sectioned and stained with luxol fast blue. Multiple microscopic images were made of representative samples of each nerve. Each myelinated axon was outlined by hand using ImageJ (Schneider et al. 2012) with the bounding line drawn at the outer edge of the myelin. A total of 3117 axons were identified and marked. Each image was then thresholded so that only the drawn outlines were visible. An algorithm in Mathematica (Wolfram Research Inc, Champaign, IL) using the Component Measurement function evaluated the number of axons, and the mean centroid distance (approximating the axon radius) were computed. An array of mean centroid distances was generated for each nerve. The mean, maximum, standard deviation of the centroid distances for each nerve were computed as well as the percentage of axons in each of the ranges of 0–1.5 *μ*m, 1.5-3 *μ*m, 3–4.5 *μ*m, 4.5–6 *μ*m, and >6 *μ*m were computed. A one-way ANOVA was used to highlight the relationship between the temperature at which ischemia occurs and the mean axon diameter. Post-hoc analyses as well as *t*-tests were applied to further define whether there were specific patterns of difference. In order to determine whether there was a significant linear relationship between anoxia temperature and axon diameter, a linear regression analysis was carried out with the mean axon diameter for each nerve as the dependent variable and the anoxia temperature and its square as independent variables. Spearman rank correlation analyses were used to determine if there was a significant association between the neurophysiologic parameters measured during the experiment and any of the histological variables including mean, median, maximum, and standard deviation of axon radius as well as the fraction of axons in the radius ranges specified above. Because this process involves over 200 comparisons, only correlations associated with a very small *P* value should be considered significant. Variables with a significant *P* < 0.005 correlation with any of these variables were entered into a forward stepwise multiple linear regression.

A second set of samples, exposed to anoxia with different glucose concentrations, were placed in 4% sodium cacodylate buffered glutaraldehyde and processed for electron microscopy. Briefly, the tissue was post fixed in buffered 2% osmium tetroxide, dehydrated in a graded ethanol series, infiltrated in epoxy resin with propylene oxide used as an intermediary solvent and finally embedded in full epoxy resin (LX112: Ladd Industries, Burlington, VT). Multiple blocks were prepared and trimmed with a Reichert-Jung Ultracut E ultramicrotome into 1 *μ*m thick sections. After a 1% Toluidine Blue histologic stain, thick sections were examined and areas selected for ultra-thin sectioning at approximately 70 nm with post uranyl acetate and lead citrate staining. Images were subsequently taken on a Zeiss (Zeiss Microscopy, Thornwood, NY) EM10 transmission electron microscope retrofitted with an SIA L3C digital camera (SIA, Duluth, GA) utilizing MaximDL software (Diffraction Limited, Ottawa, ON, Canada).

Both the light microscopy and electron microscopy images were analyzed qualitatively as well as quantitatively.

## Results

### Effects of glucose and anoxia

**Figure 1:**
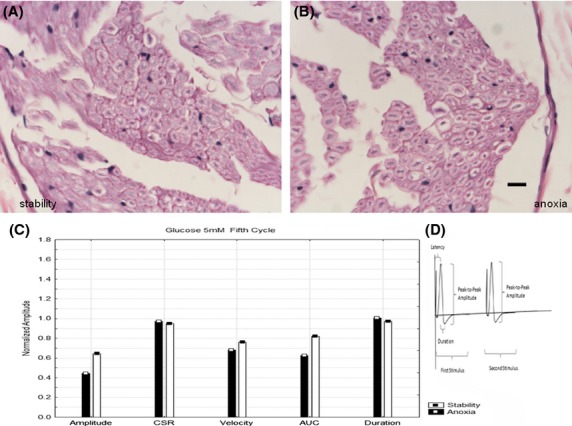
Hematoxylin and eosin stained nerve cross-sections at 60×. (A) Nerve maintained in fully oxygenated state at 37°C throughout the experiment with 5.55 mmol/L glucose showing minimal histological changes. (B) A second nerve maintained at the same temperature and glucose concentration except that it was subject to five cycles of anoxia. There are also minimal histological findings in this case. The line at the bottom of the image represents 10 microns. (C) Neurophysiologic parameters for the nerves whose images are shown above. There is decreased amplitude, area under the curve and velocity after anoxia. There is also a slight increase in duration after anoxia. CSR refers to the ratio of NAP amplitudes in a paired pulse paradigm and AUC refers to the area under the curve. (D) Illustrative example of a recorded nerve action potential and some of the measurements made.

**Figure 2:**
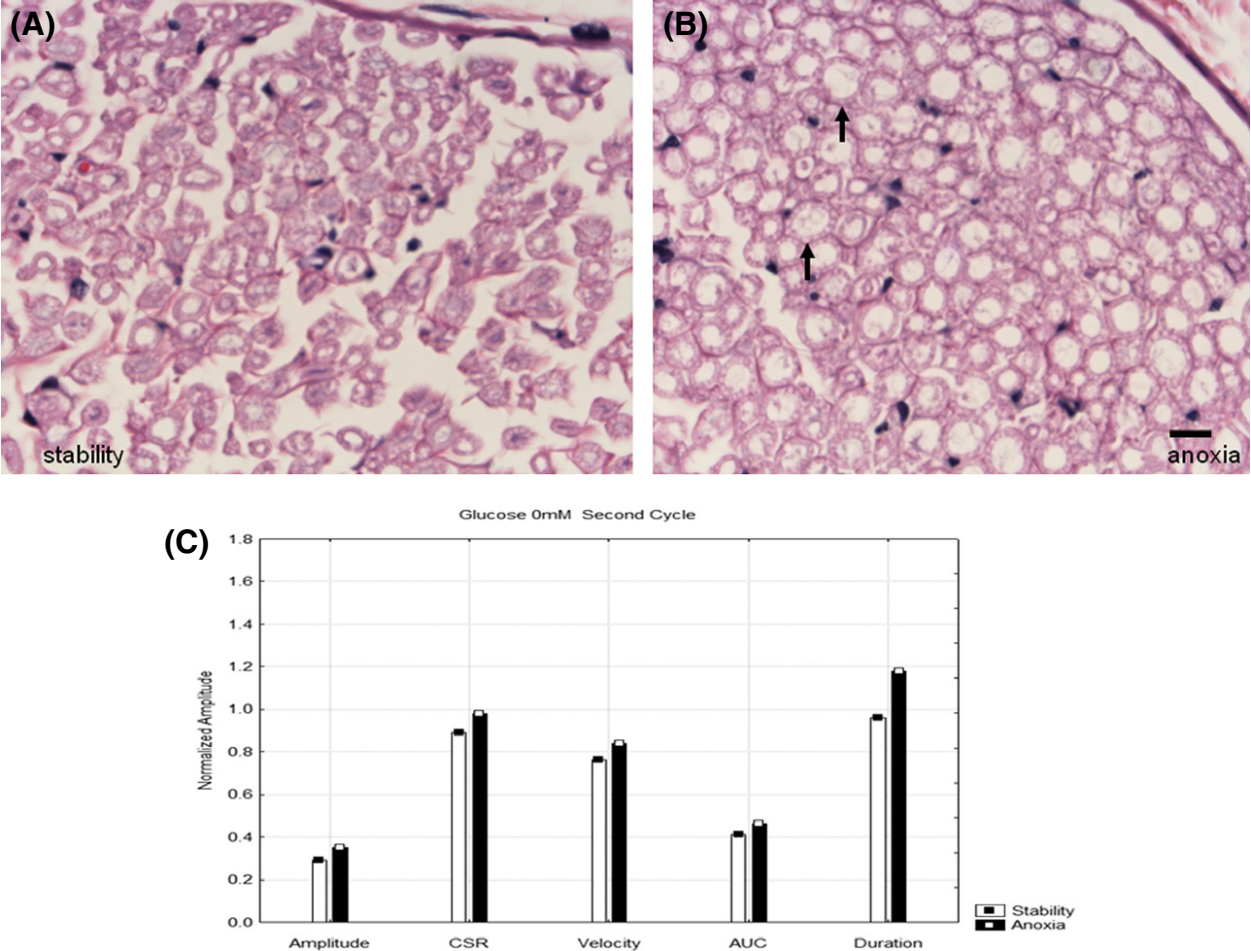
Hematoxylin and eosin stained nerve cross sections at 60×. (A) Nerve maintained in fully oxygenated state at 37°C throughout the experiment with no glucose in the perfusate. There is mild axonal swelling. (B) A second nerve exposed to five cycles of anoxia showing diffuse axonal swelling with preservation of myelin as indicated by the arrows. The line at the bottom of the image represents 10 microns. (C) Neurophysiological results in these same two nerves after the second cycle of anoxia. There were no responses in either case after the second cycle.

**Figure 3:**
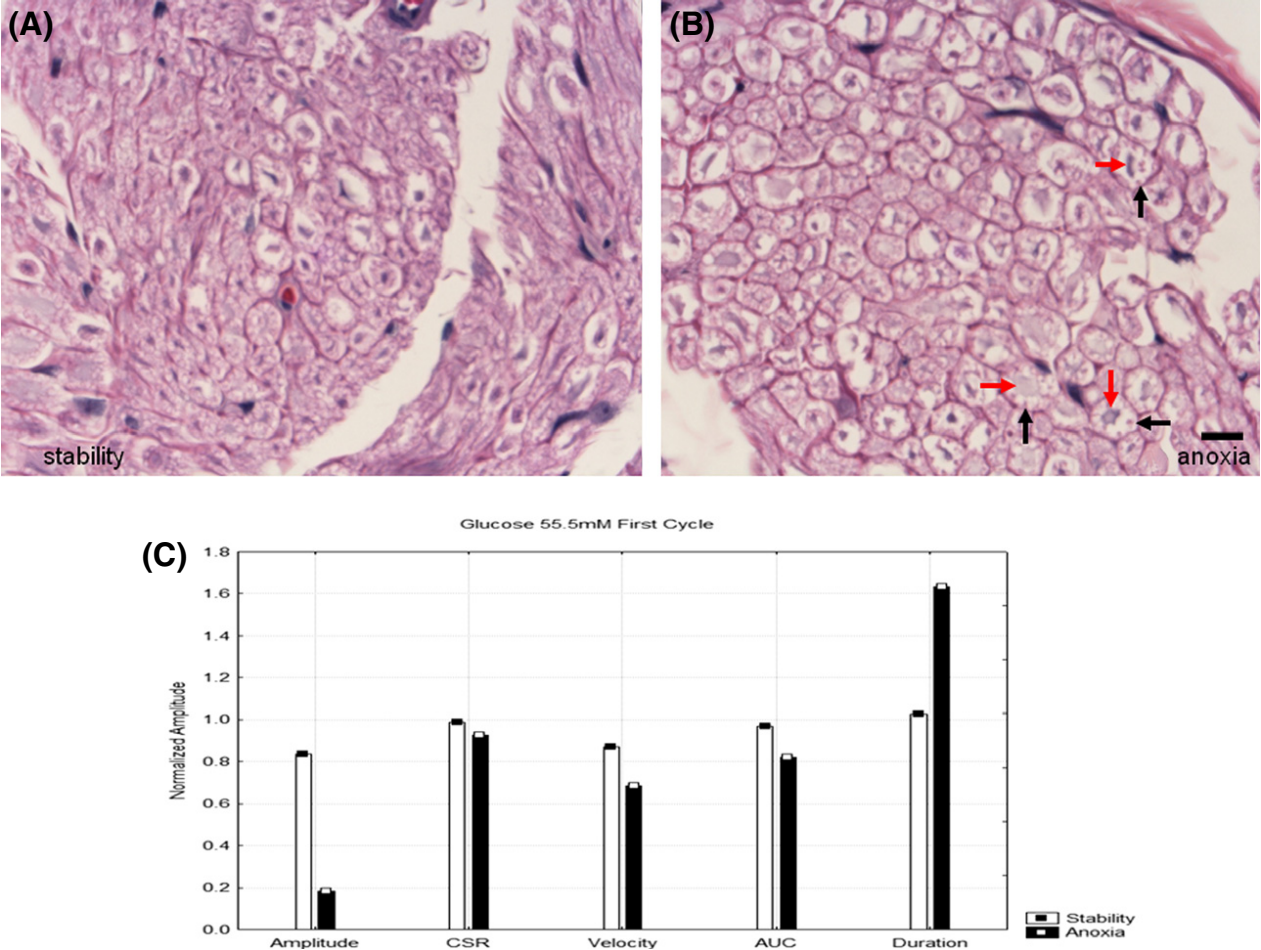
Hematoxylin and eosin stained nerve cross-sections at 60×. (A) Nerve maintained in fully oxygenated state at 37°C throughout the experiment with 55 mmol/L glucose in the perfusate. There is mild axonal swelling. (B) A second nerve exposed to five cycles of anoxia showing disruption of myelin (black arrow), with a relative preservation of axonal structure (red arrow) amid moderate axonal swelling. A notable correlation can be drawn between the cytomorphological effects (i.e. myelin disruption) seen here and that seen ultrastructurally under the same treatment conditions. The line at the bottom of the image represents 10 microns. (C) Neurophysiological results in these same two nerves after the a single cycle of anoxia. There were no responses in the nerves subjected to anoxia after the first cycle. The nerve subject to anoxia had significantly lower NAP amplitude, velocity, and area under the curve with increased durations.

Figures1A and B show H+E stained cross-sections of nerves that were maintained at 37°C with glucose 5.5 mmol/L. One (B) was subjected to five 90 min periods of anoxia and another (A) was maintained in the fully oxygenated state. There are only minor differences between these two histological cross-sections. However, the electrophysiologic parameters (Fig.1C) associated with the same nerves in Figs.1A and B shows lower amplitudes, lower velocities and lower areas under the curve as well as increased durations in the nerves that were anoxic. Previous studies (Stecker et al. 2013a,b) have demonstrated that the differences between these parameters produced by anoxia are statistically different when the results of many experiments are compared. Figure2 shows H+E stained light microscope images of nerves that were (2B) and were not (2A) exposed to anoxia at 37°C in the absence of glucose. As indicated by the arrows, there is a much greater degree of axonal swelling in the axons that were exposed to anoxia. In both cases, by the end of the experiment no NAP responses were recordable and so the neurophysiologic results shown in Fig.2C are obtained from the oxygenated period following the second period of anoxia. Although there were significant pathologic changes between the two conditions, Fig.2C demonstrates only minor neurophysiologic differences between the two nerves in the last cycle during which responses are recordable. Figure3 shows H+E stained cross-sections of nerves that were (3B) and were not (3A) exposed to anoxia with a glucose concentration of 55 mmol/L. Histologically, there is moderate axonal swelling present as well as myelin disruption that is worse after anoxia. From the neurophysiologic standpoint, there were no recordable NAP's in these nerves at the time they were removed from the in-vitro perfusion system for fixation. Figure3C shows the neurophysiologic parameters from the first cycle after anoxia, which is the last in which the anoxic and hyperglycemic nerve both have recordable responses. It is clear that the nerves subjected to anoxia have extreme reductions in the NAP amplitude and increases in duration with milder reductions in velocity.

**Figure 4:**
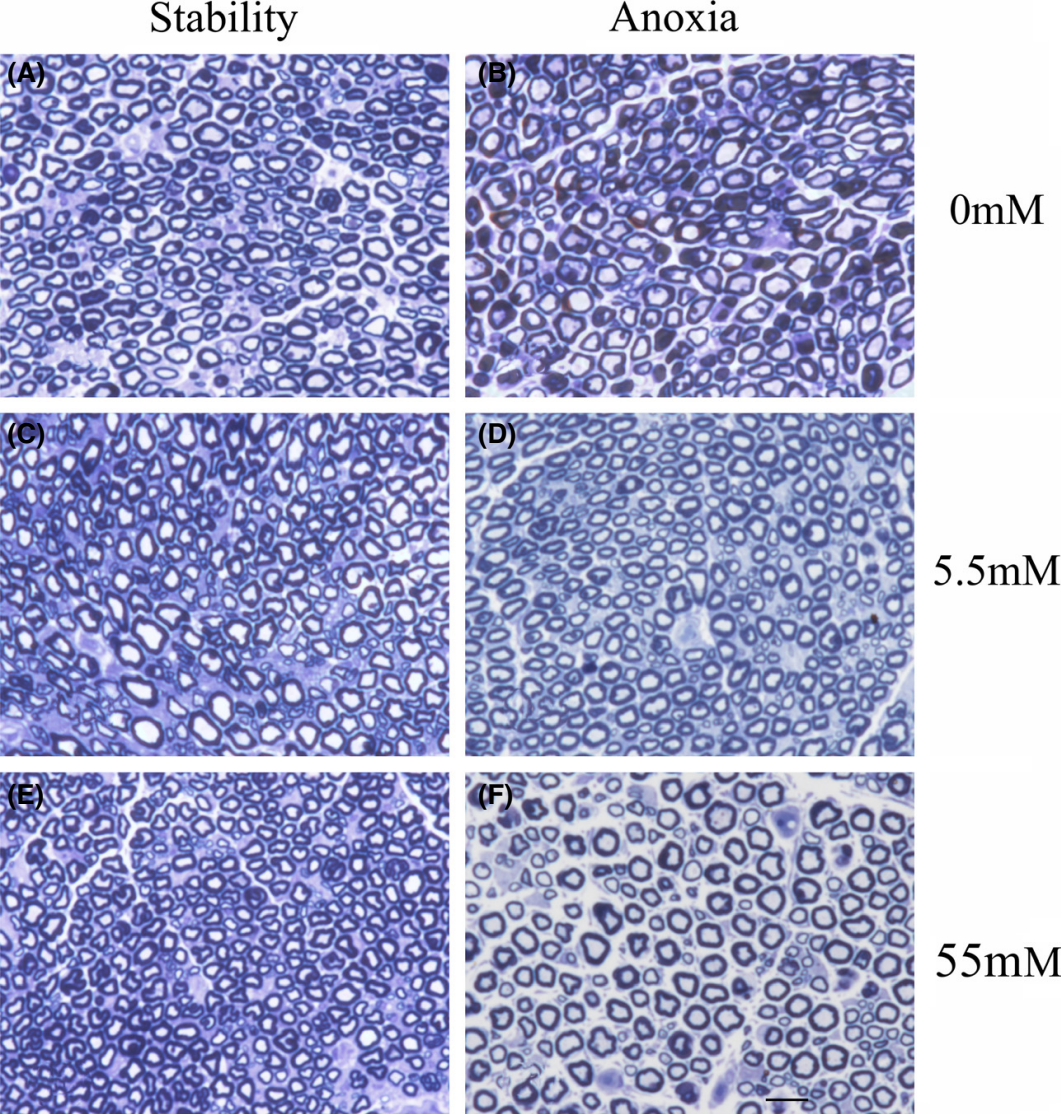
Toluidine Blue stained nerve cross-sections after resin embedding at 40×. (A) Nerve maintained in the fully oxygenated state with 0 mmol/L glucose, (B) Nerve subjected to intermittent anoxia with 0 mmol/L glucose, (C) Nerve maintained in the fully oxygenated state with 5.5 mmol/L glucose, (D) Nerve exposed to intermittent anoxia with 5.5 mmol/L glucose, (E) Nerve maintained in the continuously oxygenated state with 55 mmol/L glucose, and (F) Nerve subjected to intermittent anoxia with 55 mmol/L glucose. The shrinkage of the axons that were continuously oxygenated but exposed to hyperosmolar glucose and the enlargement of the axons of the nerves exposed to anoxia with hyperosmolar glucose or exposed to hypoglycemia is clear. The scale bar at the lower right indicates 20 microns.

**Figure 5:**
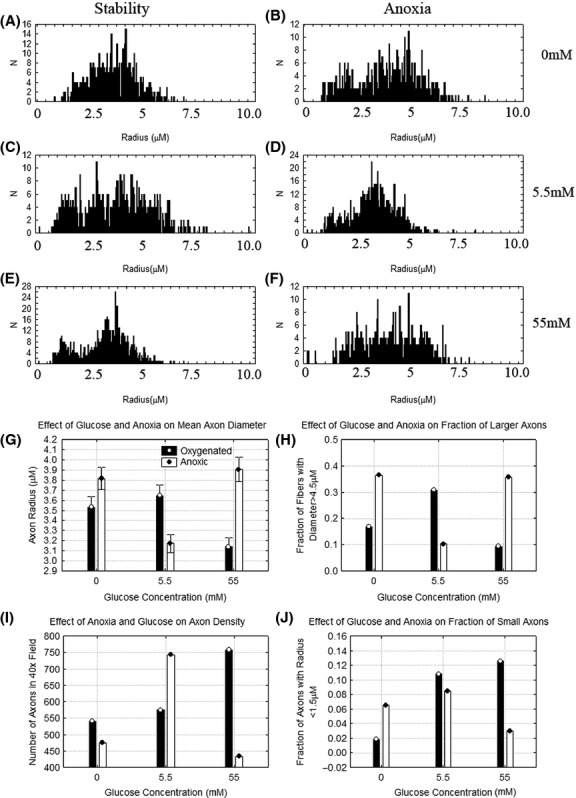
Quantitative properties of the toluidine blue images. (A) Distribution of axon radii in nerves that are continuously oxygenated at 0 mmol/L glucose, (B) Distribution of axon radii in nerves subjected to intermittent anoxia at 0 mmol/L glucose, (C) Distribution of axon radii in nerves that were continuously oxygenated at 5.5 mmol/L glucose, (D) Distribution of axon radii in nerves that were exposed to intermittent anoxia at 5.5 mmol/L glucose, (E) Distribution of axon radii in nerves that were continuously oxygenated at 55 mmol/L glucose, and (F) Distribution of axon radii in nerves that were exposed to intermittent anoxia at 55 mmol/L glucose. The error bars represent 95% confidence intervals. Changes in the (G) mean axon radius, (H) Fraction of large axons, (I) Number of axons per 40× field, and (J) Fraction of amall axons in each condition.

Because of the tissue disruption apparent in the paraffin embedded samples, resin embedded nerves stained with toluidine blue were also studied as in Fig.4. The overall anatomy is better preserved because of the resin embedding. The axons exposed to oxygen and hyperglycemia are small and irregularly shaped while those exposed to anoxia or hypoglycemia are much larger. The density of myelinated axons is lower after anoxia at 0 and 55 mmol/L glucose. Figure5 shows the distribution of myelinated axon radii in the different conditions as well as the effects of different conditions on the mean axon radius, axon density as well as the fraction of small and large axons. A factorial ANOVA demonstrates that there are significant main effects of anoxia and glucose concentration on the radii of the myelinated axons as well as a significant interaction (*F*(2, 3519) = 73.8, *P* < 0.001). The smallest myelinated axon radii are seen when the nerve is exposed to the hyperglycemic conditions and the largest when nerves are exposed to anoxia in the presence of hypo and hyperglycemic solutions. The lowest density of nerve fibers is also seen when the nerves are exposed to anoxia in the presence of hyperglycemia or hypoglycemia.

**Figure 6:**
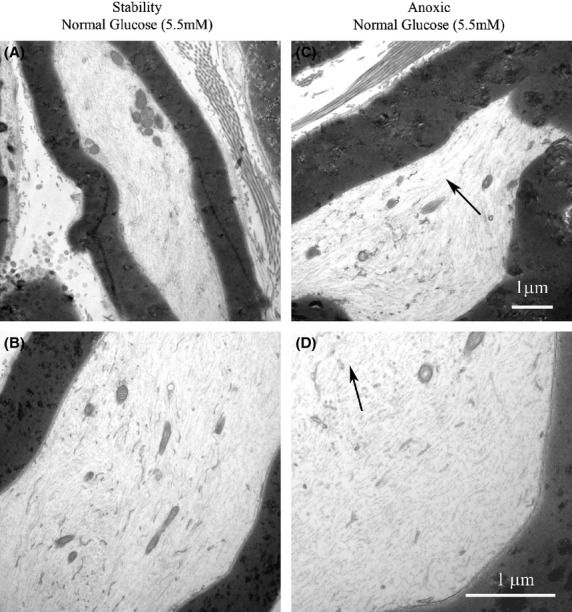
Electron micrographs of nerves that were and were not exposed to anoxia with a glucose concentration of 5.5 mmol/L. (A, B) Intact axons with well-defined axoplasm, cytoskeleton, and neurofilaments (*a* = 12,500X, *b* = 31,500X). (C, D) Degeneration of axons with loss of neurofilaments and axoplasm becoming granular (arrows) (*c* = 12,500X, *d* = 31,500X).

Figure6 shows electron microscopic images of nerves that were (6C, D) and were not (6A, B) exposed to anoxia at 37°C with glucose 5.5 mmol/L. These images show axons with loss of neurofilaments and axoplasm becoming granular in the nerves subjected to anoxia but not the nerves that were kept in the fully oxygenated state. There are not significant changes in the myelin in either condition.

**Figure 7:**
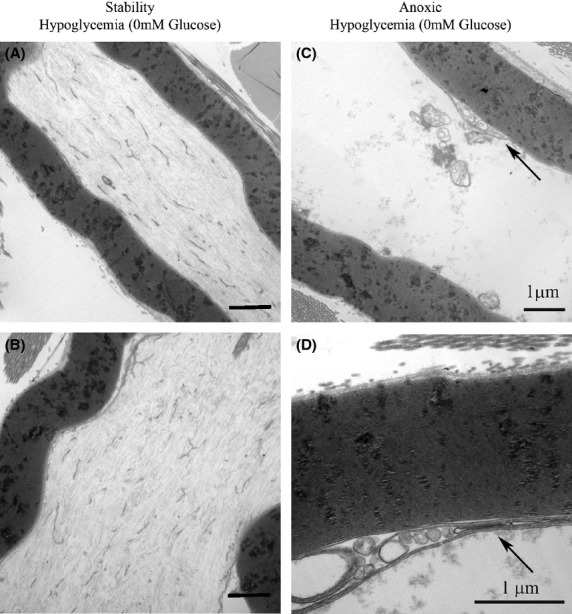
Electron micrographs of nerves that were and were not exposed to anoxia in the absence of glucose. (A, B) In the nerves not subject to anoxia, intact axons have well-defined axoplasm, cytoskeleton, and neurofilaments (*a* = 12,500X, *b* = 12,500X). (C, D) Nerves subject to anoxia in the absence of glucose demonstrate retraction of the inner loops of myelin (arrows), early axonal degeneration with loss of cytoskeleton and neurofiaments (*c* = 12,500X, *d* = 31,500X).

**Figure 8:**
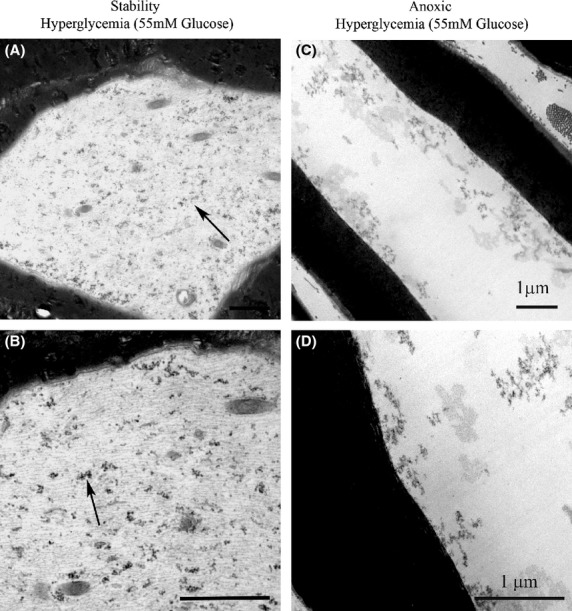
Electron micrographs of axons undergoing anoxia in the presence of 55.5 mmol/L glucose. (A, B) In the fully oxygenated state, axoplasm and cytoskeleton appear well-defined with neurofilaments present but increasing pools of glycogen (arrows) (*a* = 12,500X, *b* = 31,500X). (C, D) In the nerves subject to anoxia, degenerating axons are apparent with axoplasm showing severe disruption of the cytoskeleton and loss of neurofilaments (*c* = 12,500X, *d* = 31,500X). The contrast in images (C) and (D) was altered after acquisition in order to enhance visualization of the changes in the axoplasm.

The electron micrographs in Fig.7 show no significant changes in the nerves subject to severe hypoglycemia while being continuously oxygenated. In the nerves subject to anoxia in the absence of glucose, there is retraction of the inner loops of myelin (arrows), early axonal degeneration with loss of cytoskeleton and neurofilaments. In the nerves studied in the presence of 55 mmol/L glucose but fully oxygenated, Fig.8 shows no significant disruption of the axoplasm and cytoskeleton although pools of glycogen are noted. With exposure to anoxia, degenerating axons become apparent as well as loss of neurofilaments and disruption of the cytoskeleton. The glycogen pools noted in the fully oxygenated state are not noted with anoxia. The greatest degree of damage is evident in the case where there is anoxia in the setting of 55 mmol/L glucose. Changes are much less dramatic when anoxia occurs in the setting of normoglycemia. This is consistent with the pattern of neurophysiologic changes which are mild when anoxia occurs at normoglycemia but very severe in the setting of anoxia combined with hyperglycemia.

### Effects of hypothermia on anoxia

**Figure 9:**
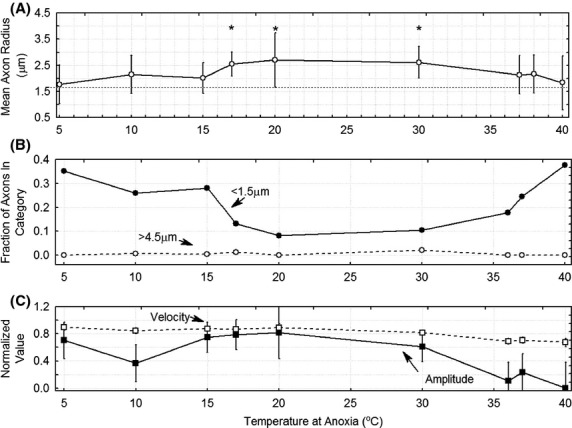
Composite figure showing: (A) Changes in mean axon radius with temperature at anoxia, (B) changes in the fraction of axons in the <1.5 mm radius and >4.5 *μ*m radius ranges, and (C) changes in the amplitude after five periods of anoxia and the velocity after two periods of anoxia. The physiologic data are from the same nerves from which the histology data is derived. The velocity after two periods of anoxia rather than after five periods of anoxia is used because there were generally no recordable NAP's after five cycles of anoxia at the higher temperatures.

Nerves that were exposed to anoxia at different temperatures were stained with luxol fast blue and the changes in axon size were analyzed using the procedure described above. Figure9A shows the mean axon radius and Fig.9B shows the fraction of small (<1.5 μm) and large (>4.5 *μ*m) axons as a function of the temperature at which anoxia occurs. The horizontal dotted line in this graph represents the mean radius of axons at 37°C that were not exposed to anoxia. A one-way ANOVA did not find a significant effect of temperature on axon radius. (*F*(8, 12) = 1.06 *P* = 0.45). However, the shape of the graph suggested that nerves undergoing anoxia at 17–30° had larger diameters than other nerves as indicated by the asterisks on the graph. A post-hoc analysis using a contrast set to 1 at other points and −1 at temperatures between 17 and 30° showed a statistically significant effect (*F*(1, 12) = 6.36 *P* = 0.03). In addition, a *t*-test comparing the radii in the 17–30°C range to the diameters at other temperatures also showed a significant difference. Regression analysis summarized in Table 2009 shows a statistically significant quadratic relationship between temperature and axon radius.

**Table 1:** Summary of the results of regression analysis describing the relationship between mean axon radius and the temperature at which anoxia occurs (*T*)

Quadratic Fit: axon radius = *a *+ *b *× *T *+ *c *× *T*^2^			
	*a*	*b*	*c*
Value	1.14	0.12	−0.0024
Std	0.44	0.043	0.001
*P*	*t*(18) = 2.62 *P* = 0.02	*t*(18) = 2.74 *P* = 0.013	*T*(18) = 2.7 *P* = 0.01

Overall: *R*^2^ = 0.294; *F*(2, 18) = 3.76; *P* < 0.04.

In order to explore the quantitative relationship between the histological and neurophysiologic parameters as temperature is changed, Spearman rank correlations were performed. This analysis showed no statistically significant correlation between any histological parameter and any physiologic parameter using the levels of significance for each test discussed above.

## Discussion

The physiology of the in-vitro sciatic nerve model is well-studied and the current study provides the anatomic correlate of the physiologic changes particularly those that occur when peripheral nerve is exposed to anoxia and different glucose concentrations.

When nerves are exposed to anoxia there is axonal swelling and disruption of the cytoskeleton as found previously in Waxman's in-vitro study of rat optic nerve (Waxman et al. 1992) and these changes are also seen in the present study of rat sciatic nerve. It is likely that these changes represent direct effects of anoxia in the peripheral nerve rather than an indirect effect due to changes in axoplasmic transport because of anoxia. In an in-vivo system where the proximal nerve segment remains in continuity with the nerve cell body, these changes could be produced by a distal blockade of axoplasmic transport as hypothesized by Nukada and Dyck (1987) and Korthals et al. (1978). However, in this in-vitro model there is no cell body and the entire nerve segment under study is uniformly affected by anoxia and so it is unlikely that there remains a significant driver for transport between one end of the nerve segment and the other.

The pathologic changes due to anoxia are minor when the nerve is maintained at normoglycemia (5.5 mmol/L) and in fact there may be a decrease in the mean axonal size. However, under conditions of hypoglycemia, nerves exposed to anoxia demonstrate clear axonal swelling when compared to nerves with fully oxygenated conditions. Nerves that were subjected to hyperglycemia (55.5 mmol/L) with anoxia showed more dramatic pathological changes than nerves that underwent anoxia at normoglycemia. In both of these conditions are associated with increases in axon diameter and reduction in the density of axons. There is also loss marked destruction of axonal structure and neurofilaments. The fact that the pathologic changes seen during anoxia in the setting of either hypoglycemia or hyperglycemia are much more dramatic than during normoglycemia is consistent with the findings of neurophysiologic studies (Stecker and Stevenson 2013). This is consistent with the fact that the clinical severity of critical illness polyneuropathy is influenced by the glucose concentration (Hermans et al. 2007, 2009; Mikaeili et al. 2012).

The relationships between axonal size, osmolarity and anoxia are important to the peripheral nerve (Maekawa et al. 2001; Galvez et al. 2003). Cells are known to adapt to hyperosmotic conditions by a number of mechanisms one of which is the induction of aldose reductase(Galvez et al. 2003) to increase the production of sorbitol. The importance of this mechanism is that it varies with cell type (Oates 2008) and may be more important in the lens than in peripheral nerve. In any case, sorbitol, because of its poor penetration through cell membranes, can be used to increase intracellular osmolarity. As the production of sorbitol utilizes NADPH, the amount of reduced glutathione may decrease and leave the cell at risk for free radical induced injury (Chung et al. 2003) especially during oxidative stress. This may be one reason why the damage with anoxia is worse in hyperglycemia.

The changes in axonal diameter with anoxia are complex. Mire et al. (1970) had demonstrated that when nerves are exposed to anoxia, there is initial swelling of axons which is followed by axonal degeneration and reduced axon size. Thus, depending on the exact timing of the anoxia in relation to the sampling and the ability of the nerve to modify metabolic pathways such as the polyol pathway to ameliorate the swelling, it is possible to see either increases or decreases in axonal diameter. This is what was observed in both the glucose/anoxia and hypothermia/anoxia experiments described in this paper. Additional studies allowing for dynamic measurements of axon size would be helpful in understanding these phenomena in more detail.

One important issue relates to the finding of intra-axonal glycogen in continuously oxygenated nerves exposed to hyperglycemia. Although most glycogen in the nervous system, under normal conditions, is located in glial cells such as Schwann cells (Brown et al. 2012), axons in the peripheral nervous system do contain glycogen phosphorylase (Pfeiffer-Guglielmi et al. 2007) which is required for glycogen breakdown. They also contain the mRNA for both glycogen phosphorylase and glycogen synthase (Pfeiffer-Guglielmi et al. 2014) and so peripheral axons likely have the capability of producing and utilizing glycogen. Intra-axonal glycogen has been previously described in peripheral axons in diabetic neuropathy (Powell et al. 1985; Orloff et al. 1988). Thomas (Thomas et al. 1980) also found glycogen in peripheral axons in older Wistar rats. In the current experiments, it is understandable that glycogen would be produced primarily in the setting of extreme hyperglycemia. If this glycogen were used during periods of anoxia to support the axon metabolically as postulated in our previous studies (Stecker and Stevenson 2015), then it would be expected that concentration of glycogen would be lower during anoxia even with hyperglycemia as we have documented.

Temperature had a dramatic effect on the response of the peripheral nerve to anoxia. Of course, lowering the temperature: decreases conduction velocity and the conditioned stimulus response possibly due to an effect on the kinetics of sodium channels (Baylor and Stecker 2009; Stecker and Baylor 2009). It also prolongs duration (Baylor and Stecker 2009; Stecker and Baylor 2009) of the NAP and produces an increase in the peak to peak amplitude at moderate temperatures. Nerves at lower temperatures during the period of anoxia were able to sustain neurophysiologic responses that were closer to baseline than nerves subjected to higher temperatures during anoxia (Stecker et al. 2013a,b). The histological changes measured by mean axon diameter were less impressive. Our data showed that axon diameters did increase with anoxia most prominently in the middle range of temperatures (17–30°C) but not the higher temperatures where the nerve was most damaged by anoxia according to the physiologic studies or at the lower temperatures where the neurophysiologic studies suggest less injury. This likely relates to the complex dynamic changes in axon size (Mire et al. 1970) discussed above. It is interesting to note that the simple measure of axon size did not correlate with any of the neurophysiologic markers studied and so these measures are complementary and contain different information.

Additional studies will be required to further understand the mechanisms underlying the anoxic damage to peripheral nerve and how that interacts with temperature and glucose concentration but this study has shown that both physiologic studies and histologic studies are important in providing a description of these processes.
